# Dose-finding designs for cumulative toxicities using multiple constraints

**DOI:** 10.1093/biostatistics/kxx059

**Published:** 2017-11-13

**Authors:** Shing M Lee, Moreno Ursino, Ying Kuen Cheung, Sarah Zohar

**Affiliations:** 1 Department of Biostatistics, Mailman School of Public Health, Columbia University, 722 W. 168th St, New York, NY, USA; 2 INSERM, UMRS 1138, Team 22, CRC, University Paris 5, University Paris 6, Paris, France

**Keywords:** Late-onset toxicities, Moderate grade toxicity, Bayesian image registration, Multiple toxicity thresholds, Time-to-event continual reassessment method, Toxicity grades, Toxicity severity

## Abstract

This article addresses the concern regarding late-onset dose-limiting toxicities (DLT), moderate toxicities below the threshold of a DLT and cumulative toxicities that may lead to a DLT, which are mostly disregarded or handled in an *ad hoc* manner when determining the maximum tolerated dose (MTD) in dose-finding cancer clinical trials. An extension of the Time-to-Event Continual Reassessment Method (TITE-CRM) which allows for the specification of toxicity constraints on both DLT and moderate toxicities, and can account for partial information is proposed. The method is illustrated in the context of an Erlotinib dose-finding trial with low DLT rates, but a significant number of moderate toxicities leading to treatment discontinuation in later cycles. Based on simulations, our method performs well at selecting the dose level that satisfies both the DLT and moderate-toxicity constraints. Moreover, it has similar probability of correct selection compared to the TITE-CRM when the true MTD based on DLT only and the true MTD based on grade 2 or higher toxicities alone coincide, but reduces the probability of recommending a dose above the MTD.

## 1. Introduction

Dose-finding clinical trials are typically small studies with the goal of evaluating the safety and tolerability of a new drug or combination of drugs in humans. In cancer clinical trials, this aim is achieved by determining the maximum tolerated dose (MTD) which is defined as the maximum dose level that satisfies a constraint based on a pre-specified target probability of dose-limiting toxicity (DLT) (Storer, 1989). Numerous methods have been proposed to estimate the MTD. Among them are algorithmic designs such as the 3+3 design, and model-based designs such as the continual reassessment method (CRM, O’Quigley *and others*, 1990)). These methods assign doses in a sequential manner after each cohort of patients based on a binary outcome, presence or absence of a DLT during a fixed pre-specified evaluation window. Even though toxicity data is collected during the entire treatment period on an ordinal scale from 0 to 5 based on the National Cancer Institute Common Terminology Criteria for Adverse Events (NCI-CTCAE) (National Cancer Institute, 2009), a DLT is generally defined as any grade 3 or higher toxicity, or dose reductions and discontinuations in the first one or two monthly cycles of treatment. This definition of a DLT which is based on the chemotherapy paradigm, only takes into account severe toxicities shortly after the initiation of treatment. New anticancer therapies such are targeted therapies and immunotherapies are administered for longer periods of time. Thus, they can lead to late-onset toxicities, and long lasting moderate toxicities resulting in dose reductions and discontinuations. A recent paper by Lee *et al.* showed a case-example using data from dose-finding trials of a targeted therapy, and reported that the DLT rates over extended cycles was 50%, higher than the conventional target DLT rate in the range of 20–33% (Lee *and others*, 2016). In current practice, dose reductions and discontinuations after the initial cycles are often stipulated in the protocol with no regard to the dose-allocation or dose-determination method, and in some instances dose reductions that occur after several cycles of treatment, and late-onset toxicities, are not taken into account in the final recommendation of the MTD (Margolin *and others*, 2001). However, in the context of novel cancer treatments, it is important to take these into account to ensure that the dose level identified is tolerable when considering longer periods of administration.

Consider, for example the dose-finding trial of erlotinib, a targeted cancer therapy, in combination with pemetrexed in patients with non-small cell lung cancer published by Ranson *and others* (2010). This trial was designed to evaluate five dose levels for up to six cycles of treatment. However, a DLT was defined as a grade 3 or 4 toxicity, any treatment-related toxicity leading to dose reduction or interruption for erlotinib in cycle 1, any treatment-related toxicity leading to delayed administration of pemetrexed in cycle 2, treatment-related death and any laboratory abnormality in the first cycle leading to dose reduction. A total of 20 patients were enrolled in the first four dose levels, with DLTs being observed in three patients at dose level 4. However, 19 out of the 20 patients experienced treatment-related toxicities and 6 withdrew from the combination prior to the end of the 6 cycles (2 experienced toxicities that led to treatment withdrawal at dose level 2, 2 experienced toxicities that led to discontinuation of pemetrexed, and 2 refused further treatment). Thus, while DLTs were not observed until dose level 4, indications of intolerability were present in dose level 2 with 2 of 6 patients needing treatment withdrawal. The recommended dose selected was dose level 3 based on the first two cycles of treatment, given that the dose reductions and treatment withdrawals at dose level 2 were observed in later cycles.

Several methods have been proposed for the inclusion of late-onset DLTs and separately for including information on gradations of toxicity severity. Cheung and Chappell (2000) introduced the time-to-event continual reassessment method (TITE-CRM) which allows for the inclusion of late-onset DLTs. Braun *and others* (2005, 2007) proposed methods that take into account a patient’s entire sequence of administrations. Liu and Braun (2009) proposed a method that constrains the probability of DLT for all patients to increase monotonically with both dose and number of administrations received. All these methods, consider toxicity as a binary outcome. Separately, several methods have been proposed to include information on toxicity severity in the estimation of the MTD based on complete observations. Van Meter *and others* (2011, 2012) proposed incorporating toxicity severity using proportional odds or continuation ratio models. Lee *et al.* proposed the continual reassessment method with multiple constraints (CRM-MC) which allows for the specification of various toxicity thresholds (Lee *and others*, 2011; Cheng and Lee, 2015). Another method has been proposed using longitudinal ordinal outcomes where the MTD is defined as the dose level associated to a DLT rate per cycle (Paoletti *and others*, 2015; Doussau *and others*, 2013). Furthermore, methods have been proposed for summarizing grades and types of toxicity into a continuous or ordinal toxicity severity score (Bekele and Thall, 2004; Yuan *and others*, 2007; Lee *and others*, 2012; Ezzalfani *and others*, 2013), and methods have been proposed to estimate the dose level associated with a target level of a toxicity score (Bekele and Thall, 2004; Yuan *and others*, 2007; Ivanova and Kim, 2009; Ezzalfani *and others*, 2013).

The aim of our work is to propose a method that can take into account partial information on both DLTs, and moderate toxicities, in order to avoid dose reduction or temporary treatment discontinuation over an extended number of cycles. In this setting, MTD is defined as the dose that satisfies not only a pre-specified DLT constraint, but also a pre-specified moderate-toxicity constraint, given a specified toxicity evaluation window that can encompass various cycles to account for late-onset and cumulative toxicities. To achieve this, we have extended both the TITE-CRM (Cheung and Chappell, 2000) and the CRM-MC (Lee *and others*, 2011; Cheng and Lee, 2015), introducing the notion of time to first moderate toxicity, as well as, time to first DLT. The new model is described in Section 2. We present the simulation studies based on the motivating example in Section 3 and end with a discussion in Section 4.

## 2. Methods

### 2.1. Model specification

Suppose we are interested in estimating the dose level associated with a target DLT probability $p_{\rm DLT}$, where $D=\{ d_1,d_2,\ldots,d_K\}$ are the $K$ doses of interest. However, we are also interested in controlling the rate of moderate toxicities, and thus we specify a target grade 2 or higher rate of $p_{\rm MT}$. Let $Z$ be an ordinal toxicity outcome, where $Z=0$ if the patient does not experience a toxicity, $Z=1$ if the patient experiences moderate toxicity without a DLT and $Z=2$ if the patient experiences a DLT. Note that these categories are mutually exclusive. If in some instances, the definition of DLT ($Z=2$) includes some grade 2 toxicities then those grade 2 toxicities would not be included in the definition of moderate toxicity ($Z=1$). Moreover, let $T_{\rm MT}$ be the time to the manifestation of the first moderate toxicity, $T_{\rm DLT}$ be the time to the manifestation of the first DLT and $c$ be the patient’s follow-up time. Then, the toxicity outcome, can be redefined as follows, $Y=0$ if $T_{\rm MT}>c$, $Y=1$ if $T_{\rm MT}\leq c$ and $T_{\rm DLT}>c$, and $Y=2$ if $T_{\rm DLT} \leq c$. That is, $Y=0$ if a toxicity has not been observed yet, $Y=1$ if only a moderate grade toxicity has been observed thus far, but no DLTs and $Y=2$ if a DLT has been observed.

Let $\mbox{Pr}(Z \geq t |x)$ denote the tail probability of $Z$ given dose $x$, the MTD, $\theta$, can be defined as the maximum dose that satisfies both pre-specified toxicity constraints, $\mbox{Pr}(Z \geq 1 \vert x) \leq p_{\rm MT}$ and $\mbox{Pr}(Z \geq 2 \vert x) \leq p_{\rm DLT}$, in terms of $Z$. That is,

(2.1) \begin{eqnarray*} \theta = \arg \max_x \left \{ \mbox{Pr}(Z \geq t_l|x) \leq p_l, l=1,2 \right \}\!, \end{eqnarray*}

where $t_1 < t_2$ are the pre-specified toxicity thresholds of 1 and 2, respectively and $p_1 \,{=}\, p_{\rm MT} \,{>}\, p_2 \,{=} p_{\rm DLT} >0$ are their respective target probabilities. To estimate the MTD, consider a generic working model,

\begin{eqnarray*}
\mbox{Pr}(Z \geq t_l \vert x) = F_l(x; \beta), \quad l=1,2,
\end{eqnarray*}

where $\beta=(\beta_1, \beta_2)^T$ is the unknown parameter.

Assuming that the maximum toxicity assessment time frame is $M$ and taking into account the censoring information,

\begin{eqnarray*}
P(Y=0|x) &=& P(T_{\rm MT}> c \vert x) = 1 -P(T_{\rm MT} \leq c \vert T_{\rm MT} \leq M, x) P(Z \geq 1 \vert x) \\
&=& 1- w_{T_{\rm MT}}(c) F _1(x;\beta)\\
P(Y=1|x) &=& P(T_{\rm MT} \leq c \vert T_{\rm MT} \leq M, x) P(Z \geq 1 \vert x)-P(T_{\rm DLT}\leq c \vert T_{\rm DLT} \leq M, x) P(Z=2 \vert x)\\
&=& w_{T_{\rm MT}}(c) F_1(x;\beta) - w_{T_{\rm DLT}}(c)F_2(x;\beta)\\
P(Y=2|x) &=& P(T_{\rm DLT}\leq c \vert T_{\rm DLT} \leq M, x) P(Z=2 \vert x)= w_{T_{\rm DLT}}(c) F_2(x;\beta), \\
\end{eqnarray*}

where $ w_{T_{\rm MT}}(c)= P(T_{\rm MT} \leq c | T_{\rm MT} \leq M, x)$ and $w_{T_{\rm DLT}}(c)=P(T_{\rm DLT}\leq c \vert T_{\rm DLT}\leq M, x)$. Thus, the likelihood of the observed data at time $c$ after $n$ patients are enrolled is:

\begin{align*}
L(\beta)&=\prod_{i=1}^n \left [ 1- w_{T_{\rm MT} i}(c) F_1(x_{[i]};\beta ) \right]^{I(Y_i=0)}
\left [w_{T_{\rm DLT} i}(c) F_2(x_{[i]};\beta) \right] ^{I(Y_i=2)} \\
&\qquad{} \left [w_{T_{\rm MT} i}(c) F_1(x_{[i]};\beta)- w_{T_{\rm DLT} i}(c)F_2(x_{[i]};\beta) \right]^{I(Y_i=1)}.
\end{align*}

### 2.2. Dose assignment

The model parameter $\beta$ can be estimated using likelihood or Bayesian methods. If the maximum likelihood estimate $\hat \beta_n$ exists, operationally the recommended dose for the $(n+1)$-th patient is the minimum of the dose levels that minimizes the distance to each pre-specified target, that is,

$$
x(n+1)= \min \{ {\rm argmin}_{x\in D} \vert F_1(x; \hat\beta_{n}) - p_{\rm MT}\vert,{ \rm argmin}_{x\in D}
\vert F_2(x; \hat\beta_{n})-p_{\rm DLT}\vert \}.
$$

For example, if after $n$ patients, the dose level such that $F_1(x; \hat\beta_{n})$ is closest to $p_{\rm MT}$ is dose level 3 and the dose level such that $F_2(x; \hat\beta_{n})$ is closest to $p_{\rm DLT}$ is dose level 4, the $(n+1)$-th patient will be assigned dose level 3. It is worth noting that $\hat \beta_n$ does not exist until all possible values of $Z$ are observed. Thus, to estimate $\beta$ using the maximum likelihood estimate, we take a staged approach which has been previously described in Cheng and Lee (2015) and is also illustrated in detail in Section 2.3.

If Bayesian methods are used for the estimation of $\beta$, the marginal posterior distribution of the dose levels that minimizes the distance to each pre-specified target can be obtained and the dose given to the next patient is,

$$ x(n+1) = \arg \min_{x\in D} \vert x - \min (\tilde{x}_{\rm MT}, \tilde{x}_{\rm DLT}) \vert,$$

where $\tilde{x}_{\rm MT}$ indicates the marginal posterior median dose level based on grade 2 or higher toxicities and $\tilde{x}_{\rm DLT}$ the marginal posterior median dose level based on DLTs. For example, if after $n$ patients, the marginal posterior median dose level based on grade 2 or higher toxicities is dose level 3 and the posterior median dose level based on DLTs is dose level 4, the $n+1$ patient will be assigned dose level 3. This is the same dose assignment approach used for the CRM with multiple constraints (Lee *and others*, 2011). It should be noted that if Bayesian methods are used, it can be used from the beginning of the trial.

### 2.3. Simulation study

Using the setting of the erlotinib and pemetrexed trial, we assume five dose levels ($K=5$). Given that, we are interested in controlling for both the rate of DLT and moderate toxicities, two toxicity constraints are imposed. The first constraint is that the probability of grade 2 or higher toxicities is less than 0.50, that is, $P(Z \geq 1 \vert x) \leq p_{\rm MT}=0.50$. The second constraint is that the probability of DLT is less than 0.25, $P(Z=2 \vert x) \leq p_{\rm DLT}=0.25$. Even though the sample size for the study was 20, we consider a sample size of 24 given that only four dose levels were evaluated in the original trial. We also consider a sample size of 40 to evaluate the impact of sample size. The evaluation window is six cycles of treatment. We assume an empiric working model as proposed in Cheng and Lee (2015) and in line with the conventional empiric model often used for the CRM. Thus, $F_1(x; \beta) = x^{\beta_1}$ and $ F_2(x; \beta) = x^{\beta_1 +\beta_2} $. The skeleton was selected based solely on the DLT constraint using the indifference interval ($\delta$) calibration approach by Lee and Cheung (2009). Given five dose levels, a target DLT rate of 0.25, and assuming the MTD is dose level 3 and an empiric dose-toxicity working model, the $\delta$ value for sample sizes of 24 and 40 are 0.07 and 0.06, respectively. Given the minor differences in the $\delta$ values, a $\delta$ of 0.06 which corresponds to 0.06, 0.14, 0.25, 0.38, 0.50, was used for both sample sizes. Moreover, in line with the original TITE-CRM which showed that the linear weight function yielded similar operating characteristics compared to more complicated weight functions, we assume that the weight functions are linear, that is, $w_{T_{MT}} (c) = w_{T_{DLT}} (c) = c/6$. Dose skipping was not allowed.

Two-thousand simulations were performed using both likelihood and Bayesian approaches. { For the likelihood approach a three-stage design was implemented whereby cohorts of three were used in the first-stage until the occurrence of a grade 2 or higher toxicity. If only grade 2 or lower toxicities were observed, the TITE-CRM using the first constraint, $P(Z \geq 1 | x)\leq 0.50$, was applied. If the occurrence of DLTs and no toxicities were observed, but no grade 2 toxicities, the TITE-CRM using DLT as outcome was applied. Once full heterogeneity was achieved, that is, at least one patient was observed with each possible value of the outcome at the end of six cycles, the proposed method was invoked. The second stage was skipped if full heterogeneity was achieved in the first-stage. Moreover, for our simulations, if in the first cohort grade 2 toxicities (but no DLT) were observed for all patients, we assigned the same dose for the next cohort. If in the first cohort DLTs were observed for all patients, we stopped the trial.

For the Bayesian approach, we considered two distinct parametrizations and associated prior distributions. In the first case each parameter $\beta_l$, where $l=1, 2$, is parametrized in the exponential form, that is $\beta_l = {\rm e}^{\gamma_l}$, where each $\gamma_l$ is assumed to have independent normal distribution with zero mean and standard deviation of 1.34 as prior distribution. This is the conventional parametrization and prior used for the empiric working function in the dfcrm package in R (Cheung, 2013; R Core Team, 2013). In the second case, we use the parametrization proposed for the CRM with multiple constraints (Lee *and others*, 2011), and assume independent exponential distributions with a rate of 1 as the prior distribution for each $\beta_l$ in line with the CRM convention (O’Quigley *and others*, 1990; Yuan *and others*, 2007). Based on these priors, we can use Markov Chain Monte Carlo (MCMC) to obtain the marginal posterior distribution of the maximum dose level which satisfies the individual constraints $P(Z \geq 1 \,{\vert}\, \tilde{x}_{\rm MT}) \leq p_{\rm MT}$ and $P(Z \geq 2 \vert \tilde{x}_{\rm DLT}) \leq p_{\rm DLT}$ and in both cases assign doses based on the approach previously mentioned in Section 2.2. The MCMC were performed using R 3.2.1 (R Core Team, 2013) with rstan version 2.6.0. The same skeletons were used for both likelihood and Bayesian approaches.

To simulate cumulative toxicities over time we utilized a discrete-time Markov process whereby a transition matrix $P$ was specified. The transition matrix is upper triangular given that it represents the maximal toxicity grade which can only worsen with time. All patients start at baseline without any toxicities and transition to states with maximal toxicities of grade 2 (moderate toxicity) or DLTs. Each step of the Markov process represents a toxicity assessment visit at the end of a cycle of treatment. Given that we are interested in three categories of outcomes a $3\times3$ transition matrix is specified for each dose level. The maximum toxicity assessment time frame of six cycles is used to define the MTD, that is, the probability of maximal grades at state M, $P^6$, is used for the definition of the MTD. Various different simulation scenarios are presented. The transition matrices used for generating the various scenarios are displayed in supplementary material available at *Biostatistics* online. In addition, for each data set patients are assumed to arrive either at a fixed rate of six patients in six cycles (i.e. one patient per cycle) or assuming that the number of patients follows a Poisson distribution with the same rate of the fixed accrual rate. In these cases, patient responses remained unchanged and only the entry time into the trial is altered. For each dose assignment, when using the proposed method, patients who have not reached the maximum time frame of six cycles are included in the likelihood based on the simulated outcome at a time $c$ less than six with a respective linear weight of $c/6$ based on their observed follow-up.

Given that there are no comparison methods, we compared our results to the benchmark for the CRM with multiple constraints using complete observations after six cycles to assess the performance our method (Cheung, 2014). The benchmark is a theoretical design which provides an accuracy upper-bound given our design objective. It is only theoretical because it requires complete toxicity profiles which are not observed in practice. For the benchmark, the complete outcome distribution for all dose levels was specified based on the various scenarios, complete toxicity profiles were simulated based on the sample size of the trial, and the sample proportions for each of the dose levels were calculated using the simulated toxicity profiles for each constraint. The recommended dose level based on the benchmark for the CRM-MC with complete observations is the minimum of the dose levels that minimizes the distance between the sample proportions and each pre-specified target. Moreover, for the scenarios in which the two constraints coincide, we compared our results to those of the TITE-CRM using only the DLT constraint. Under those scenarios, the MTD for the proposed method and the TITE-CRM is the same. The Bayesian TITE-CRM was used with an empiric working function, a $N(0, 1.34)$ prior for the model parameter, and the same skeleton mentioned above, 0.06, 0.14, 0.25, 0.38, 0.50. This is the conventional prior used for the empiric working function in the dfcrm package in R (Cheung, 2013; R Core Team, 2013)

## 3. Results

### 3.1 Simulation trial

**Fig. 1.**
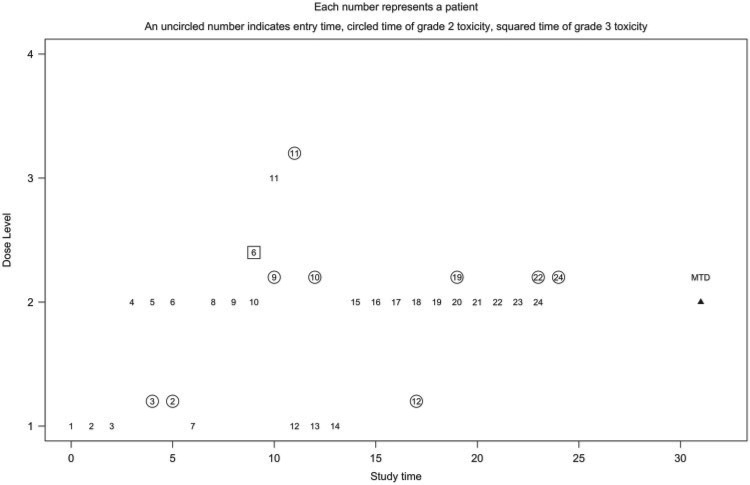
Simulated trial using the proposed method with likelihood estimation.

We illustrate the proposed method using a single simulated trial with 24 patients and the design parameters specified in Section 2.3. Figure 1 displays the dose levels assigned and the toxicity outcomes for the 24 patients in the trial. The *x*-axis is the study time in months. The *y*-axis is the dose level at which the patient was treated. Each number represents a patient’s entry time into the study using a fixed accrual of one patient per month. A circled number indicates the time to first moderate toxicity and a squared number indicates the time to first DLT. This example is generated based on scenario 10 where the true MTD is dose level 2 with the constraint for grade 2 or higher toxicities being the limiting one. The parameters are estimated using likelihood estimation with a three-staged design with cohort sizes of three in the first stage. Thus, the first three patients were assigned dose level 1. The dose was escalated to dose level 2 for the next cohort (patients 4, 5, and 6) with no toxicities being observed by the third month. The second and third patient both experienced their first moderate toxicity by the fifth month and the second stage was invoked whereby the seventh patient was assigned using the TITE-CRM to dose level 1. The dose was escalated again to dose level 2 for patients 8 and 9. By the ninth month, full heterogeneity was present with the sixth patient having had their first DLT, using the proposed method, patient 10 was treated at dose level 2. With a new occurrence of moderate toxicity for patient 9 in the 10th month, the dose was still escalated to dose level 3 for patient 11 who had a moderate toxicity the first month. Thus, the dose was de-escalated to dose level 1 for patient 12. By the 12th month, patient 10 had their first moderate toxicity in dose level 2, and thus patients 13 and 14 remained at dose level 1. The remaining 10 patients were all assigned dose level 2 with two occurrences of moderate toxicity, one for patient 12 in dose level 1 and one for patient 19 in dose level 2. Two additional moderate toxicities were observed during follow-up for patients 22 and 24. At the end of the trial, the method recommended dose level 2 as the MTD, with grade 2 or higher toxicities reported in 6 out of 16 patients (38%) and one DLT reported.

### 3.2. Simulation results

**Fig. 2.**
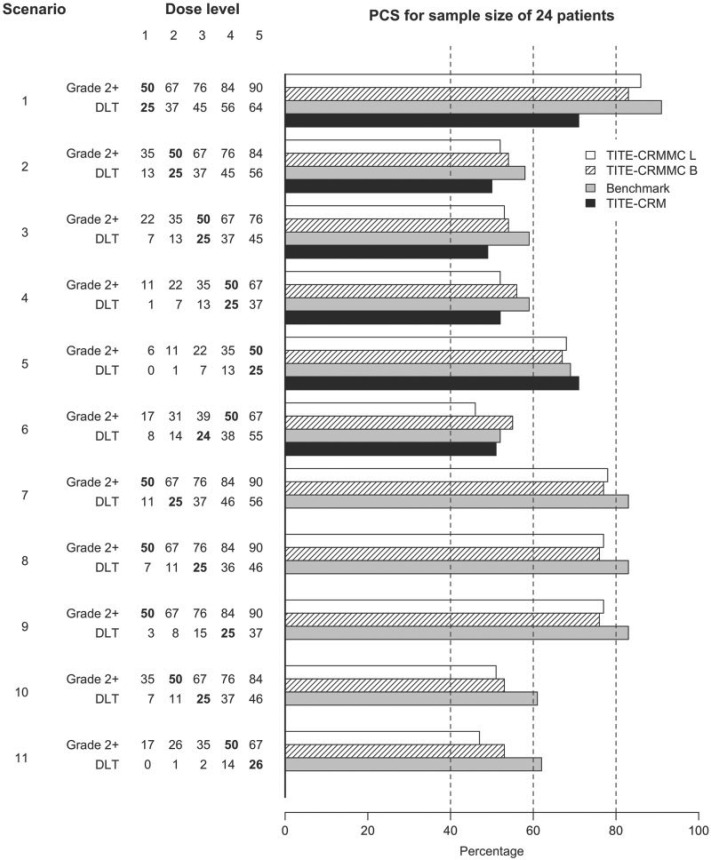
Probability of correct selection (PCS) of the proposed method, referred as TITE-CRMMC, 24 patients. L stands for likelihood estimation using staged approach. B stands for Bayesian. Grade 2+ indicates the probability of grade 2 or higher toxicity. DLT indicates the probability of DLT. The true MTD based on each constraint is specified in bold.

**Fig. 3.**
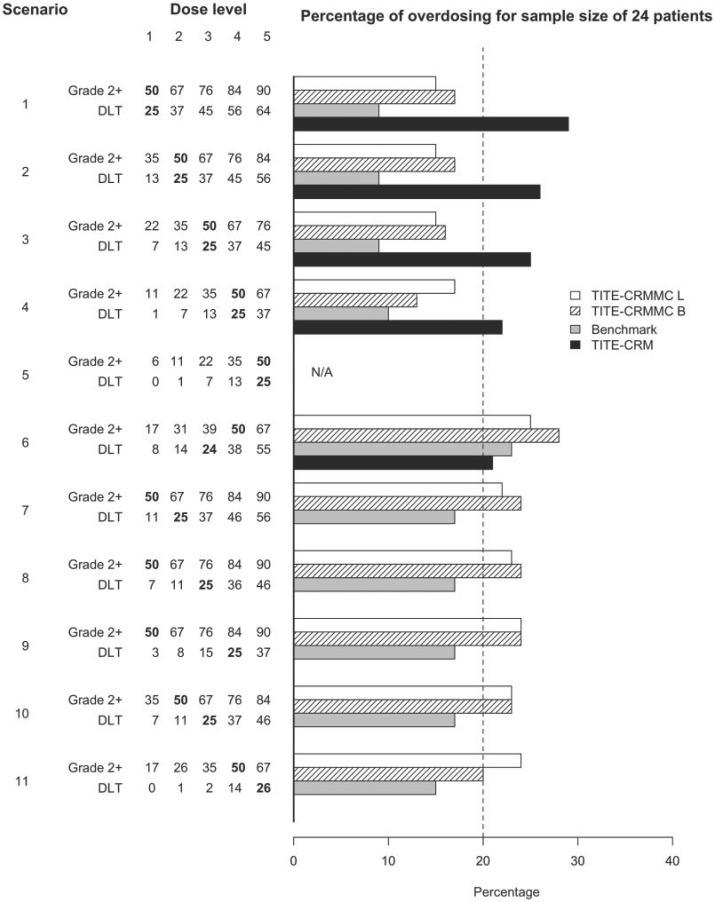
Probability of recommending a dose above the MTD for the proposed method, referred to as TITE-CRMMC, 24 patients. L stands for likelihood estimation using staged approach. B stands for Bayesian. Grade 2+ indicates the probability of grade 2 or higher toxicity. DLT indicates the probability of DLT. The true MTD based on each constraint is specified in bold. N/A indicates not applicable since the true MTD is the highest dose level.

The simulation results for a sample sizes of 24 are displayed in Figures 2 and 3 when data are generated using a fixed accrual rate of 1 patient per cycle. Figure 2 displays the probability of recommending the true MTD, and Figure 3 displays the probability of recommending a dose above the true MTD. The top five scenarios in each figure display scenarios where the true MTD based on grade 2 or higher toxicity and DLT constraints coincide. The bottom six scenarios display scenarios were the two constraints do not coincide. In scenarios 7–11, the true MTD based on grade 2 or higher toxicities is a lower dose level than that based on DLT. The white bars represent the proposed method, referred to as the TITE-CRMMC, using likelihood estimation. The stripe bars represent the proposed method using Bayesian estimation. The gray bars represent the results for the benchmark and the solid black bars the results using the TITE-CRM. For the scenarios when the two constraints coincided, the TITE-CRM with multiple constraints had a much higher probability of correct selection (PCS) compared to the TITE-CRM when the true MTD was dose level one (0.86 and 0.83 for the likelihood and Bayesian approaches, respectively versus 0.71) and similar performance, within 0.05, when the true MTD was higher. Moreover, in all of these scenarios having multiple constraints substantially reduced the probability of selecting a dose above the true MTD compared to the TITE-CRM. The difference depended on the true MTD and, as expected, decreased when the true MTD was a higher dose level. Thus, when the primary and secondary constraints coincide, the TITE-CRM with multiple constraints has similar PCS compared to the TITE-CRM, but is more conservative. The PCS for the TITE-CRM with multiple constraints is slightly worse than the PCS of its benchmark, but still within 0.01 to 0.08 across all scenarios. When the limiting constraint was the DLT constraint, suggesting that the additional constraint on moderate toxicities was unnecessary, the performance of the proposed method using likelihood estimation was slightly worse compared to the TITE-CRM, but using Bayesian estimation the performance was slightly better in terms of PCS. However, the methods were not as conservative and recommended doses above the true MTD more frequently than the TITE-CRM (differences within 0.04 and 0.07). The TITE-CRM with multiple constraints performed well at selecting the true MTD when the constraint regarding grade 2 or higher toxicities was the limiting constraint. While it had lower PCS compared to when both constraints coincided (within 0.10 for the likelihood approach and within 0.07 for the Bayesian approach for all scenarios), in some instances the benchmark also had lower PCS, indicating that these are harder scenarios. The performance was also not affected by the number of dose levels between the true MTD based on the DLT constraint and the constraint based on grade 2 or higher toxicities as indicated by scenarios 7–9. Generally, the performance of the likelihood staged approach was similar to that using Bayesian estimation starting from the first patient. The likelihood approach performed slightly worse when the true MTD did not coincide and it was one of the higher dose levels (scenarios 6 and 11).

The PCS and the probability of recommending a dose above the true MTD improved for all methods using a sample size of 40. The relative performance of the methods was similar compared to using a sample size of 24. With the larger sample size, the results for the proposed method using likelihood and Bayesian estimation were more similar, especially in the few scenarios were there were slight differences using a sample size of 24 such as scenarios 6 and 11. The results based on a fixed rate of entry and assuming that the number of patients follows a Poisson distribution with the same accrual rate were very similar. The tables displaying the simulation results, as well as, the results for simulations assuming the number of patients enrolled in the study follows a Poisson distribution and for a sample size of 40 are available as supplementary material at *Biostatistics* online. The tables also display information on the number of patients that were assigned before full heterogeneity was observed to invoke both constraints. Using likelihood estimation and the staged approach, the median number of patients before full heterogeneity was observed ranged between 10 and 22 depending on the true probabilities of toxicities and the entry time. This could explain why under some scenarios the performance of the TITE-CRM with multiple constraints is similar to the TITE-CRM. Moreover, the two parametrizations used along with the Bayesian estimation yielded identical results (data not shown). Thus, only the results using the normal prior are displayed.

## 4. Discussion

In this article, we propose a method for including partial information when imposing multiple constraints. This method is an extension of the TITE-CRM that includes in the model both time to first DLT and time to a first secondary event, such as grade 2 or higher toxicity. By including outcome and corresponding follow-up time prior to reaching the maximum time evaluation window in the modeling framework using a weighted likelihood during the conduct of the trial the method allows for the toxicity assessment window to be extended to evaluate late-onset as well as cumulative moderate and DLT which are disregarded when using current methods with assessment windows of one or two cycles. Thus, dose recommendations during the trial are based on partial information at a given time point. However, the final recommendation is based on complete observations and only depends on the failure time distribution through in-trial dose assignments, similar to the TITE-CRM.

Both moderate and late-onset toxicities are important to consider when estimating the MTD because they can lead to dose-reductions, dose-discontinuations, and intolerability over time. Indeed, the general assumption for cytostatic agents, such as erlotinib, is that the more cycles a patient is treated before progression the better it is. Moreover, unless a patient progresses, treatment should be given until a desired success outcome. This is why controlling both acute and cumulative toxicity is highly important. In this work, we have tried to achieve this goal by proposing a method that not only takes into account DLTs that occur during the first one or two cycles, but also DLTs and moderate toxicities occurring after long-term administration. This is illustrated in our motivating example where there was a need to control for the rates of both DLT and moderate toxicities, which did not necessarily occur in the first couple of treatment cycles. While a simple solution would be to redefine DLT based on a longer evaluation window and including moderate toxicities, extending the evaluation window can substantially increase the duration of a trial when using complete observations. Furthermore, including moderate toxicity in the definition of DLT and using TITE-CRM may identify an overly conservative dose when using conventional target DLT rates of 0.25% to 0.33%.

The method has good performance characteristics with sample sizes similar to those used for a single DLT constraint and performs better than the TITE-CRM with a single DLT constraint in the case that both the primary and secondary constraints for the individual toxicity constraints coincide. Moreover, as observed with the CRM with multiple constraints, having multiple constraints can help reduce the probability of selecting doses above the MTD while yielding a similar PCS. The method can be easily implemented in practice using either Bayesian or likelihood estimation. While the performance of the two are similar, the likelihood estimation may be preferred by clinicians given that the specification of a prior is not required and it is easier computationally. However, it also requires a staged approach based on the heterogeneity of responses.

Moreover, the proposed method can easily be extended to account for three constraints, a constraint for grade 2 or higher toxicities, a constraint for DLT, and potentially a constraint for life-threatening and deadly toxicities, or using a continuous or ordinal toxicity severity outcome with three relevant severity thresholds. The MTD is then defined as the maximum dose that satisfies all three constraints. From the practical point of view, there seems to be little use in applying more than three constraints. Given that the definition of mild toxicities is much more subjective and mild toxicities are very prevalent, imposing a constraint for mild toxicities should be carefully considered. Future dose-finding clinical trials should take into account cumulative toxicity at least in the final recommendation of the MTD if it cannot be done during the dose allocation process. Currently, there is a big gap between dose recommendation and everyday practice in which dose adjustment and modifications are routinely done according to toxicities observed after the first cycle. Therefore, early phase clinical trials should align with clinical practice, and stop focusing on the first cycle only, especially in the context of new anticancer treatments.

## 5. Software

Software in the form of R code is available on GitHub and can be requested from the corresponding author (sml2114@cumc.columbia.edu).

## Supplementary Material

Supplementary Materials
