# Supplementary material for: Dose-finding designs for cumulative toxicities using multiple constraints
Source: Biostatistics. 2017 Nov 13;20(1):17–29. doi: 10.1093/biostatistics/kxx059 (PMC6296314; doi:10.1093/biostatistics/kxx059)
Supplement: Supplementary Materials [file kxx059_supplementary_materials.pdf]

Biostatistics (2017), **0**, 0, *pp.* 1–6
doi:10.1093/biostatistics/"Supplementary Materials SM Lee et al R1 Biostatistics"

Supplementary Materials for Dose-finding designs for cumulative toxicities using multiple constraints

SHING M. LEE*

Department of Biostatistics, Columbia University, 722 W. 168th St, New York, New York, USA.

SML2114@cumc.columbia.edu

MORENO URSINO

INSERM, UMRS 1138, team 22, CRC, University Paris 5, University Paris 6, Paris, France.

YING KUEN CHEUNG**

Department of Biostatistics, Columbia University, 722 W. 168th St, New York, New York, USA.

SARAH ZOHAR**

INSERM, UMRS 1138, team 22, CRC, University Paris 5, University Paris 6, Paris, France.

[]

*To whom correspondence should be addressed
**Contributed equally as co-senior authors

© The Author 2017. Published by Oxford University Press. All rights reserved. For permissions, please e-mail: journals.permissions@oup.com

Table 1. *Transition matrices used for the simulation scenarios 1-11.*

Scenario	Dose Level 1	2	3	4	5
Scenario 1	$\begin{bmatrix} 0.89 & 0.08 & 0.03 \\ 0 & 0.87 & 0.13 \\ 0 & 0 & 1 \end{bmatrix}$	$\begin{bmatrix} 0.83 & 0.12 & 0.05 \\ 0 & 0.85 & 0.15 \\ 0 & 0 & 1 \end{bmatrix}$	$\begin{bmatrix} 0.79 & 0.14 & 0.07 \\ 0 & 0.84 & 0.16 \\ 0 & 0 & 1 \end{bmatrix}$	$\begin{bmatrix} 0.74 & 0.16 & 0.1 \\ 0 & 0.82 & 0.18 \\ 0 & 0 & 1 \end{bmatrix}$	$\begin{bmatrix} 0.68 & 0.19 & 0.13 \\ 0 & 0.8 & 0.20 \\ 0 & 0 & 1 \end{bmatrix}$
Scenario 2	$\begin{bmatrix} 0.93 & 0.06 & 0.01 \\ 0 & 0.89 & 0.11 \\ 0 & 0 & 1 \end{bmatrix}$	$\begin{bmatrix} 0.89 & 0.08 & 0.03 \\ 0 & 0.87 & 0.13 \\ 0 & 0 & 1 \end{bmatrix}$	$\begin{bmatrix} 0.83 & 0.12 & 0.05 \\ 0 & 0.85 & 0.15 \\ 0 & 0 & 1 \end{bmatrix}$	$\begin{bmatrix} 0.79 & 0.14 & 0.07 \\ 0 & 0.84 & 0.16 \\ 0 & 0 & 1 \end{bmatrix}$	$\begin{bmatrix} 0.74 & 0.16 & 0.1 \\ 0 & 0.82 & 0.18 \\ 0 & 0 & 1 \end{bmatrix}$
Scenario 3	$\begin{bmatrix} 0.96 & 0.03 & 0.01 \\ 0 & 0.96 & 0.04 \\ 0 & 0 & 1 \end{bmatrix}$	$\begin{bmatrix} 0.93 & 0.06 & 0.01 \\ 0 & 0.89 & 0.11 \\ 0 & 0 & 1 \end{bmatrix}$	$\begin{bmatrix} 0.89 & 0.08 & 0.03 \\ 0 & 0.87 & 0.13 \\ 0 & 0 & 1 \end{bmatrix}$	$\begin{bmatrix} 0.83 & 0.12 & 0.05 \\ 0 & 0.85 & 0.15 \\ 0 & 0 & 1 \end{bmatrix}$	$\begin{bmatrix} 0.79 & 0.14 & 0.07 \\ 0 & 0.84 & 0.16 \\ 0 & 0 & 1 \end{bmatrix}$
Scenario 4	$\begin{bmatrix} 0.98 & 0.02 & 0 \\ 0 & 0.97 & 0.03 \\ 0 & 0 & 1 \end{bmatrix}$	$\begin{bmatrix} 0.96 & 0.03 & 0.01 \\ 0 & 0.96 & 0.04 \\ 0 & 0 & 1 \end{bmatrix}$	$\begin{bmatrix} 0.93 & 0.06 & 0.01 \\ 0 & 0.89 & 0.11 \\ 0 & 0 & 1 \end{bmatrix}$	$\begin{bmatrix} 0.89 & 0.08 & 0.03 \\ 0 & 0.87 & 0.13 \\ 0 & 0 & 1 \end{bmatrix}$	$\begin{bmatrix} 0.83 & 0.12 & 0.05 \\ 0 & 0.85 & 0.15 \\ 0 & 0 & 1 \end{bmatrix}$
Scenario 5	$\begin{bmatrix} 0.99 & 0.01 & 0 \\ 0 & 0.98 & 0.02 \\ 0 & 0 & 1 \end{bmatrix}$	$\begin{bmatrix} 0.98 & 0.02 & 0 \\ 0 & 0.97 & 0.03 \\ 0 & 0 & 1 \end{bmatrix}$	$\begin{bmatrix} 0.96 & 0.03 & 0.01 \\ 0 & 0.96 & 0.04 \\ 0 & 0 & 1 \end{bmatrix}$	$\begin{bmatrix} 0.93 & 0.06 & 0.01 \\ 0 & 0.89 & 0.11 \\ 0 & 0 & 1 \end{bmatrix}$	$\begin{bmatrix} 0.89 & 0.08 & 0.03 \\ 0 & 0.87 & 0.13 \\ 0 & 0 & 1 \end{bmatrix}$
Scenario 6	$\begin{bmatrix} 0.97 & 0.02 & 0.01 \\ 0 & 0.90 & 0.10 \\ 0 & 0 & 1 \end{bmatrix}$	$\begin{bmatrix} 0.94 & 0.05 & 0.01 \\ 0 & 0.85 & 0.15 \\ 0 & 0 & 1 \end{bmatrix}$	$\begin{bmatrix} 0.92 & 0.06 & 0.02 \\ 0 & 0.75 & 0.25 \\ 0 & 0 & 1 \end{bmatrix}$	$\begin{bmatrix} 0.89 & 0.06 & 0.065 \\ 0 & 0.7 & 0.3 \\ 0 & 0 & 1 \end{bmatrix}$	$\begin{bmatrix} 0.83 & 0.09 & 0.08 \\ 0 & 0.65 & 0.35 \\ 0 & 0 & 1 \end{bmatrix}$
Scenario 7	$\begin{bmatrix} 0.89 & 0.09 & 0.2 \\ 0 & 0.98 & 0.02 \\ 0 & 0 & 1 \end{bmatrix}$	$\begin{bmatrix} 0.83 & 0.12 & 0.05 \\ 0 & 0.96 & 0.04 \\ 0 & 0 & 1 \end{bmatrix}$	$\begin{bmatrix} 0.79 & 0.14 & 0.07 \\ 0 & 0.92 & 0.08 \\ 0 & 0 & 1 \end{bmatrix}$	$\begin{bmatrix} 0.74 & 0.17 & 0.09 \\ 0 & 0.89 & 0.11 \\ 0 & 0 & 1 \end{bmatrix}$	$\begin{bmatrix} 0.68 & 0.20 & 0.12 \\ 0 & 0.86 & 0.14 \\ 0 & 0 & 1 \end{bmatrix}$
Scenario 8	$\begin{bmatrix} 0.89 & 0.10 & 0.1 \\ 0 & 0.98 & 0.02 \\ 0 & 0 & 1 \end{bmatrix}$	$\begin{bmatrix} 0.83 & 0.15 & 0.02 \\ 0 & 0.98 & 0.02 \\ 0 & 0 & 1 \end{bmatrix}$	$\begin{bmatrix} 0.79 & 0.16 & 0.05 \\ 0 & 0.96 & 0.04 \\ 0 & 0 & 1 \end{bmatrix}$	$\begin{bmatrix} 0.74 & 0.18 & 0.08 \\ 0 & 0.94 & 0.06 \\ 0 & 0 & 1 \end{bmatrix}$	$\begin{bmatrix} 0.68 & 0.21 & 0.11 \\ 0 & 0.92 & 0.08 \\ 0 & 0 & 1 \end{bmatrix}$
Scenario 9	$\begin{bmatrix} 0.89 & 0.11 & 0 \\ 0 & 0.98 & 0.02 \\ 0 & 0 & 1 \end{bmatrix}$	$\begin{bmatrix} 0.83 & 0.16 & 0.01 \\ 0 & 0.98 & 0.02 \\ 0 & 0 & 1 \end{bmatrix}$	$\begin{bmatrix} 0.79 & 0.19 & 0.02 \\ 0 & 0.96 & 0.04 \\ 0 & 0 & 1 \end{bmatrix}$	$\begin{bmatrix} 0.74 & 0.21 & 0.05 \\ 0 & 0.96 & 0.04 \\ 0 & 0 & 1 \end{bmatrix}$	$\begin{bmatrix} 0.68 & 0.25 & 0.07 \\ 0 & 0.92 & 0.8 \\ 0 & 0 & 1 \end{bmatrix}$
Scenario 10	$\begin{bmatrix} 0.93 & 0.06 & 0.01 \\ 0 & 0.98 & 0.2 \\ 0 & 0 & 1 \end{bmatrix}$	$\begin{bmatrix} 0.89 & 0.09 & 0.02 \\ 0 & 0.98 & 0.02 \\ 0 & 0 & 1 \end{bmatrix}$	$\begin{bmatrix} 0.83 & 0.12 & 0.05 \\ 0 & 0.96 & 0.04 \\ 0 & 0 & 1 \end{bmatrix}$	$\begin{bmatrix} 0.79 & 0.14 & 0.07 \\ 0 & 0.92 & 0.8 \\ 0 & 0 & 1 \end{bmatrix}$	$\begin{bmatrix} 0.74 & 0.16 & 0.1 \\ 0 & 0.9 & 0.1 \\ 0 & 0 & 1 \end{bmatrix}$
Scenario 11	$\begin{bmatrix} 0.97 & 0.03 & 0.00 \\ 0 & 0.99 & 0.01 \\ 0 & 0 & 1 \end{bmatrix}$	$\begin{bmatrix} 0.95 & 0.05 & 0 \\ 0 & 0.98 & 0.02 \\ 0 & 0 & 1 \end{bmatrix}$	$\begin{bmatrix} 0.93 & 0.07 & 0 \\ 0 & 0.98 & 0.02 \\ 0 & 0 & 1 \end{bmatrix}$	$\begin{bmatrix} 0.89 & 0.09 & 0.02 \\ 0 & 0.96 & 0.04 \\ 0 & 0 & 1 \end{bmatrix}$	$\begin{bmatrix} 0.83 & 0.13 & 0.04 \\ 0 & 0.93 & 0.07 \\ 0 & 0 & 1 \end{bmatrix}$

Table 2. *Operating characteristic of the TITE-CRMMC (Time to Event Continual Reassessment Method with Multiple Constraints) when the primary and secondary constraints coincide, 24 patients. L stands for Likelihood Estimation using Staged Approach. B stands for Bayesian. The true MTD based on each constraint is specified in bold.*

	Dose Level					n starting		
	1	2	3	4	5	0.5q	0.25q	0.75q
					Scenario 1			
Probability of Grade 2 or higher toxicity	**0.50**	0.67	0.76	0.84	0.90			
Probability of DLT	**0.25**	0.37	0.45	0.56	0.64			
TITE-CRMMC L	**86**	14	1	0	0	10	9	12
TITE-CRMMC L/ Poisson	**86**	13	1	0	0	11	9	14
TITE-CRMMC B	**83**	16	1	0	0			
TITE-CRMMC B/ Poisson	**82**	16	1	0	0			
Benchmark	**91**	9	0	0	0			
TITE-CRM	**71**	25	4	0	0			
TITE-CRM/ Poisson	**70**	26	4	0	0			
					Scenario 2			
Probability of Grade 2 or higher toxicity	0.35	**0.50**	0.67	0.76	0.84			
Probability of DLT	0.13	**0.25**	0.37	0.45	0.56			
TITE-CRMMC L	34	**52**	14	1	0	10	9	12
TITE-CRMMC L/ Poisson	33	**53**	14	1	0	12	10	15
TITE-CRMMC B	29	**54**	16	1	0			
TITE-CRMMC B/ Poisson	29	**55**	15	1	0			
Benchmark	32	**58**	9	0	0			
TITE-CRM	24	**50**	22	4	0			
TITE-CRM/ Poisson	23	**49**	23	4	0			
					Scenario 3			
Probability of Grade 2 or higher toxicity	0.22	0.35	**0.50**	0.67	0.76			
Probability of DLT	0.07	0.13	**0.25**	0.37	0.45			
TITE-CRMMC L	3	29	**53**	14	1	13	11	16
TITE-CRMMC L/ Poisson	3	31	**52**	14	1	14	12	18
TITE-CRMMC B	2	28	**54**	15	1			
TITE-CRMMC B/Poisson	2	27	**55**	15	0			
Benchmark	2	29	**59**	9	0			
TITE-CRM	2	24	**49**	22	3			
TITE-CRM/Poisson	2	23	**50**	22	3			
					Scenario 4			
Probability of Grade 2 or higher toxicity	0.11	0.22	0.35	**0.50**	0.67			
Probability of DLT	0.01	0.07	0.13	**0.25**	0.37			
TITE-CRMMC L	0	2	29	**52**	17	16	13	19
TITE-CRMMC L/Poisson	0	3	27	**54**	16	17	14	20
TITE-CRMMC B	0	2	29	**56**	13			
TITE-CRMMC B/Poisson	0	2	29	**55**	14			
Benchmark	0	2	29	**59**	10			
TITE-CRM	0	2	24	**52**	22			
TITE-CRM/ Poisson	0	2	24	**53**	22			
					Scenario 5			
Probability of Grade 2 or higher toxicity	0.06	0.11	0.22	0.35	**0.50**			
Probability of DLT	0	0.01	0.07	0.13	**0.25**			
TITE-CRMMC L	0	0	3	29	**68**	19	16	22
TITE-CRMMC L/ Poisson	0	0	2	28	**70**	20	17	23
TITE-CRMMC B	0	0	2	31	**67**			
TITE-CRMMC B/ Poisson	0	0	2	31	**66**			
Benchmark	0	0	2	29	**69**			
TITE-CRM	0	0	2	27	**71**			
TITE-CRM/Poisson	0	0	3	27	**70**			

Table 3. *Operating characteristic of the TITE-CRMMC (Time to Event Continual Reassessment Method with Multiple Constraints) when the limiting constraint is the probability of DLT for scenario 6 and Grade 2 or higher toxicities for scenarios 7-11, 24 patients. L stands for Likelihood Estimation using Staged Approach. B stands for Bayesian. The true MTD based on each constraint is specified in bold.*

	Dose Level					n starting		
	1	2	3	4	5	0.5q	0.25q	0.75q
					Scenario 6			
Probability of Grade 2 or higher toxicity	0.17	0.31	0.39	**0.50**	0.67			
Probability of DLT	0.08	0.14	**0.24**	0.38	0.55			
TITE-CRMMC L	3	26	**46**	22	3	14	12	17
TITE-CRMMC L/ Poisson	4	27	**45**	21	3	15	12	19
TITE-CRMMC B	1	17	**55**	27	1			
TITE-CRMMC B/ Poisson	1	19	**52**	26	2			
Benchmark	3	22	**52**	22	1			
TITE-CRM	3	25	**51**	20	1			
TITE-CRM/ Poisson	3	25	**50**	20	1			
					Scenario 7			
Probability of Grade 2 or higher toxicity	**0.50**	0.67	0.76	0.84	0.9			
Probability of DLT	0.11	**0.25**	0.37	0.46	0.56			
TITE-CRMMC L	**78**	21	1	0	0	11	9	13
TITE-CRMMC L/ Poisson	**78**	21	1	0	0	12	10	16
TITE-CRMMC B	**77**	22	2	0	0			
TITE-CRMMC B/Poisson	**78**	20	2	0	0			
Benchmark	**83**	16	1	0	0			
					Scenario 8			
Probability of Grade 2 or higher toxicity	**0.50**	0.67	0.76	0.84	0.90			
Probability of DLT	0.07	0.11	**0.25**	0.36	0.46			
TITE-CRMMC L	**77**	21	2	0	0	13	10	19
TITE-CRMMC L/ Poisson	**78**	20	1	0	0	14	11	20
TITE-CRMMC B	**76**	22	2	0	0			
TITE-CRMMC B/ Poisson	**76**	21	2	0	0			
Benchmark	**83**	16	1	0	0			
					Scenario 9			
Probability of Grade 2 or higher toxicity	**0.50**	0.67	0.76	0.84	0.90			
Probability of DLT	0.03	0.08	0.15	**0.25**	0.37			
TITE-CRMMC L	**77**	22	2	0	0	19	12	>24
TITE-CRMMC L/ Poisson	**79**	20	1	0	0	20	13	>24
TITE-CRMMC B	**76**	22	2	0	0			
TITE-CRMMC B/ Poisson	**76**	21	2	0	0			
Benchmark	**83**	16	1	0	0			
					Scenario 10			
Probability of Grade 2 or higher toxicity	0.35	**0.50**	0.67	0.76	0.84			
Probability of DLT	0.07	0.11	**0.25**	0.37	0.46			
TITE-CRMMC L	26	**51**	22	1	0	13	10	16
TITE-CRMMC L/ Poisson	24	**52**	22	2	0	14	11	18
TITE-CRMMC B	24	**53**	21	2	0			
TITE-CRMMC B/ Poisson	24	**54**	20	2	0			
Benchmark	22	**61**	16	1	0			
					Scenario 11			
Probability of Grade 2 or higher toxicity	0.17	0.26	0.35	**0.50**	0.67			
Probability of DLT	0	0.01	0.02	0.14	**0.26**			
TITE-CRMMC L	0	3	25	**47**	24	20	16	>24
TITE-CRMMC L/ Poisson	0	4	27	**44**	26	22	17	>24
TITE-CRMMC B	0	3	25	**53**	20			
TITE-CRMMC B/ Poisson	0	3	25	**54**	18			
Benchmark	0	2	20	**62**	15			

Table 4. *Operating characteristic of the TITE-CRMMC (Time-to-Event Continual Reassessment Method with Multiple Constraints) when primary and secondary constraints coincide, 40 patients. L stands for Likelihood Estimation using Staged Approach. B stands for Bayesian. The true MTD based on each constraint is specified in bold.*

	Dose Level					n starting		
	1	2	3	4	5	0.5q	0.25q	0.75q
Scenario 1								
Probability of Grade 2 and higher toxicity	**0.50**	0.67	0.76	0.84	0.90			
Probability of DLT	**0.25**	0.37	0.45	0.56	0.64			
TITE-CRMMC L	**90**	10	0	0	0	10	9	12
TITE-CRMMC L / Poisson	**90**	10	0	0	0	11	9	14
TITE-CRMMC B	**89**	11	0	0	0			
TITE-CRMMC B / Poisson	**88**	12	0	0	0			
Benchmark	**95**	5	0	0	0			
TITE-CRM	**78**	20	1	0	0			
TITE-CRM/ Poisson	**76**	22	2	0	0			
Scenario 2								
Probability of Grade 2 or higher toxicity	0.35	**0.50**	0.67	0.76	0.84			
Probability of DLT	0.13	**0.25**	0.37	0.45	0.56			
TITE-CRMMC L	25	**66**	9	0	0	10	9	12
TITE-CRMMC L/ Poisson	24	**67**	10	0	0	12	10	15
TITE-CRMMC B	24	**66**	11	0	0			
TITE-CRMMC B/ Poisson	22	**68**	10	0	0			
Benchmark	25	**69**	5	0	0			
TITE-CRM	17	**62**	20	1	0			
TITE-CRM/ Poisson	17	**61**	20	2	0			
Scenario 3								
Probability of Grade 2 or higher toxicity	0.22	0.35	**0.50**	0.67	0.76			
Probability of DLT	0.07	0.13	**0.25**	0.37	0.45			
TITE-CRMMC L	0	26	**63**	10	0	13	11	16
TITE-CRMMC L/ Poisson	1	26	**63**	10	0	14	12	18
TITE-CRMMC B	0	24	**65**	10	0			
TITE-CRMMC B /Poisson B	0	23	**66**	10	0			
Benchmark	0	25	**69**	5	0			
TITE-CRM	0	18	**61**	20	0			
TITE-CRM/ Poisson	0	18	**62**	19	1			
Scenario 4								
Probability of Grade 2 or higher toxicity	0.11	0.22	0.35	**0.50**	0.67			
Probability of DLT	0.01	0.07	0.13	**0.25**	0.37			
TITE-CRMMC L	0	0	23	**66**	11	16	13	19
TITE-CRMMC L/Poisson	0	0	23	**66**	11	17	14	20
TITE-CRMMC B	0	0	23	**68**	8			
TITE-CRMMC B/ Poisson	0	0	24	**66**	10			
Benchmark	0	0	25	**69**	5			
TITE-CRM	0	0	19	**63**	18			
TITE-CRM/ Poisson	0	1	18	**63**	18			
Scenario 5								
Probability of Grade 2 or higher toxicity	0.06	0.11	0.22	0.35	**0.50**			
Probability of DLT	0	0.01	0.07	0.13	**0.25**			
TITE-CRMMC L	0	0	1	23	**76**	19	16	22
TITE-CRMMC L/ Poisson	0	0	1	22	**78**	20	17	23
TITE-CRMMC B	0	0	0	26	**74**			
TITE-CRMMC B/ Poisson	0	0	0	25	**75**			
Benchmark	0	0	0	25	**75**			
TITE-CRM	0	0	0	20	**79**			
TITE-CRM/ Poisson	0	0	1	21	**79**			

Table 5. *Operating characteristic of the TITE-CRMMC (Time to Event Continual Reassessment Method with Multiple Constraints) when the limiting constraint is the probability of DLT for scenario 6 and Grade 2 or higher toxicities for scenarios 7-11, 40 patients. L stands for Likelihood Estimation using Staged Approach. B stands for Bayesian. The true MTD based on each constraint is specified in bold.*

	Dose Level					n starting		
	1	2	3	4	5	0.5q	0.25q	0.75q
				Scenario 6				
Probability of Grade 2 or higher toxicity	0.17	0.31	0.39	**0.50**	0.67			
Probability of DLT	0.08	0.14	**0.24**	0.38	0.55			
TITE-CRMMC L	1	19	**61**	19	0	14	12	17
TITE-CRMMC L/ Poisson	1	18	**62**	18	0	15	12	19
TITE-CRMMC B	0	14	**65**	20	0			
TITE-CRMMC B/ Poisson	0	15	**64**	21	0			
Benchmark	1	17	**65**	17	0			
TITE-CRM	1	20	**63**	16	0			
TITE-CRM/ Poisson	1	21	**61**	17	0			
				Scenario 7				
Probability of Grade 2 or higher toxicity	**0.50**	0.67	0.76	0.84	0.90			
Probability of DLT	0.11	**0.25**	0.37	0.46	0.56			
TITE-CRMMC L	**83**	17	0	0	0	11	9	13
TITE-CRMMC L/ Poisson	**83**	17	0	0	0	12	10	16
TITE-CRMMC B	**84**	15	0	0	0			
TITE-CRMMC B/ Poisson	**84**	16	0	0	0			
Benchmark	**89**	11	0	0	0			
				Scenario 8				
Probability of Grade 2 or higher toxicity	**0.50**	0.67	0.76	0.84	0.90			
Probability of DLT	0.07	0.11	**0.25**	0.36	0.46			
TITE-CRMMC L	**82**	17	0	0	0	13	10	19
TITE-CRMMC L/ Poisson	**84**	16	0	0	0	14	11	20
TITE-CRMMC B	**84**	16	0	0	0			
TITE-CRMMC B/ Poisson	**84**	16	0	0	0			
Benchmark	**89**	11	0	0	0			
				Scenario 9				
Probability of Grade 2 or higher toxicity	**0.50**	0.67	0.76	0.84	0.90			
Probability of DLT	0.03	0.08	0.15	**0.25**	0.37			
TITE-CRMMC L	**83**	17	0	0	0	19	12	35
TITE-CRMMC L/ Poisson	**83**	16	0	0	0	20	13	38
TITE-CRMMC B	**84**	16	0	0	0			
TITE-CRMMC B/ Poisson	**84**	16	0	0	0			
Benchmark	**89**	11	0	0	0			
				Scenario 10				
Probability of Grade 2 or higher toxicity	0.35	**0.50**	0.67	0.76	0.84			
Probability of DLT	0.07	0.11	**0.25**	0.37	0.46			
TITE-CRMMC L	17	**66**	17	0	0	13	10	16
TITE-CRMMC L/ Poisson	18	**65**	17	0	0	14	11	18
TITE-CRMMC B	19	**64**	16	0	0			
TITE-CRMMC B/ Poisson	18	**65**	16	0	0			
Benchmark	16	**73**	11	0	0			
				Scenario 11				
Probability of Grade 2 or higher toxicity	0.17	0.26	0.35	**0.50**	0.67			
Probability of DLT	0	0.01	0.02	0.14	**0.26**			
TITE-CRMMC L	0	0	19	**66**	14	20	16	28
TITE-CRMMC L/ Poisson	0	1	19	**66**	14	22	17	30
TITE-CRMMC B	0	1	19	**66**	14			
TITE-CRMMC B/ Poisson	0	1	21	**64**	14			
Benchmark	0	0	16	**73**	11			
